# Deep Learning-Based Classification of Temporal Stages of AT8-Labeled Tau Pathology After Experimental Traumatic Brain Injury

**DOI:** 10.1007/s12021-025-09763-0

**Published:** 2026-01-19

**Authors:** Guilherme José de Antunes e Sousa, Rodrigo Afonso Sá, Marcos António Spínola Monteiro Gomes, George A. Edwards, Ines Moreno-González, Ricardo José Alves de Sousa

**Affiliations:** 1 TEMA - Centre for Mechanical Technology and Automation, Department of Mechanical Engineering, University of Aveiro, Campo Universitário De Santiago, 3810-193 Aveiro, Portugal; 2 LASI—Intelligent Systems Associate Laboratory, Guimarães, Portugal; 3 Department of Physics, UA – University of Aveiro, Campo Universitário De Santiago, 3810-193 Aveiro, Portugal; 4 BCM – Baylor College of Medicine, One Baylor Plaza, Houston, TX 77030 USA; 5 Department of Cellular Biology, Genetics and Physiology, UMA – University of Malaga, Avda. Cervantes, 2. 29071, Málaga, Spain

**Keywords:** Tauopathy, Traumatic Brain Injury (TBI), AT8 immunohistochemistry, Deep learning, Convolutional neural networks (CNN), Transfer learning, Computational histopathology, Temporal classification

## Abstract

Tauopathies are characterised by a progressive accumulation of hyperphosphorylated tau. However, early and intermediate stages remain challenging to quantify due to subtle and heterogeneous morphological characteristics. This study evaluates a deep learning framework for classifying multiple temporal stages of tauopathy progression using AT8 (anti-phospho-tau antibody)-stained cortical micrographs in a controlled traumatic brain injury mouse model – an underexplored application. Three convolutional neural network (CNN) architectures were examined: a custom CNN and two transfer-learning models (InceptionV3 and DenseNet). Images were grouped into four post-injury stages: 1 day, 1 week, 1 month and 3 months. Preprocessing included normalisation, augmentation and oversampling to address imbalance. Performance was assessed using stratified k-fold cross-validation with accuracy, macro-F1, per-class F1, and one-vs-rest area under the receiver operating characteristic curve (AUC). DenseNet achieved the best overall performance (accuracy = 70.9%, macro-F1 = 0.68) with strong discrimination for the 1-week stage (F1 = 0.95). All models showed limited separability in the earliest post-injury stage (1 day), while intermediate to late stages (1–3 months) exhibited partial overlap, consistent with the progressive nature of tau accumulation. These results indicate that deep learning, particularly transfer learning, offers a scalable approach for automated temporal staging of tauopathy in preclinical histology. Although the results are based on internal cross-validation without independent animal-level identifiers or external cohorts, the proposed framework provides a reliable foundation for incorporating CNN-based analysis into digital neuropathology workflows. Larger multi-centre datasets and slide-level modelling will be required to assess generalisation and support applications in early detection, longitudinal tracking, and treatment evaluation of tau-related neurodegeneration.

## Introduction

Traumatic brain injury (TBI) is the major cause of long-term neurological impairment and raises the probability of chronic neurodegenerative diseases (Blennow et al., 2012; Gavett et al., 2011; Kovacs, 2018; Masel & DeWitt, 2010; Stern et al., 2011). According to experimental and clinical investigations, TBI can cause tau protein misfolding, phosphorylation, and aggregation, resulting in tauopathy-like neuronal and glial inclusions (Arendt et al., 2016; Jucker & Walker, 2011; Yoshiyama et al., 2007). In controlled experimental models, these changes occur in a time-structured manner: early post-injury stages typically show little or no detectable pathology, intermediate stages show increasing neuritic and somatic accumulation, and late stages result in widespread cortical involvement (Calignon et al., 2012; Clavaguera et al., 2009; Cuello et al., 2010; Edwards et al., 2020; Hoshino et al., 1998; Tran et al., 2011). These stage-specific changes occur in the transgenic animal employed in this work at four predetermined time intervals: one day, one week, one month, and three months post-injury.

AT8 immunostaining is often used in histological evaluations of tau pathology because it specifically binds phosphorylated tau to serine 202 and threonine 205. AT8 is highly selective for hydrophosphorylated tau species, labelling perisomatic aggregates, neuropil threads, and neuritic pathology (Goedert et al., 1995; Yoshiyama et al., 2007). Although commonly employed, manual staging of AT8-stained microscope images is time-consuming, subjective, and requires expert judgement. Early pathology may be subtle, but intermediate and late stages share overlapping characteristics (Hoshino et al., 1998). Variability in staining intensity, tissue processing, and field selection all compromise reproducibility (Edwards et al., 2020), emphasising the importance of automated and quantitative procedures in computational neuropathology. Importantly, AT8's molecular specificity and spatially consistent labelling make it an appropriate target for computational staging, as its signal reflects early phosphorylation events that can contain discriminative cues across time intervals.

Deep learning has emerged as an effective approach for histopathology image analysis. Convolutional neural networks (CNNs) can learn discriminative morphological features directly from raw pixel data and have been used to identify mitosis, quantify biomarkers, and perform other tissue categorisation tasks (Cireşan et al., 2013; Signaevsky et al., 2019; Tang et al., 2019). CNNs have been used to analyse whole-brain histology, detect protein aggregation, classify neurodegenerative lesions, and quantify tau load in Alzheimer's disease and related conditions (Cireşan et al., 2013; Signaevsky et al., 2019; Tang et al., 2019). CNNs have also been used in cortical histology tasks, such as automatically segmenting laminar patterns in the cerebral cortex (Wang et al., 2024). These studies show that CNNs can detect subtle microstructural patterns that manual assessment cannot; however, current computational work is primarily focused on Alzheimer's-related aggregates, binary pathology discrimination, and macroscopic neuroimaging modalities such as PET (Positron Emission Tomography) and MRI (Magnetic Resonance Imaging) (Liu et al., 2018; Zou et al., 2021). As a result, automated multistage temporal classification of TBI-induced tauopathy using high-resolution bright-field histology remains largely unexplored.

Several methodological and biological factors contribute to this gap. Tau pathology progresses along a continuum, with morphological differences between stages being primarily quantitative rather than qualitatively distinct, especially shortly after injury. AT8-stained cortical sections can vary substantially in terms of staining intensity, background texture, and tissue orientation (Edwards et al., 2020; Zanier et al., 2018), making feature extraction and model generalization challenging. The lack of slide- or subject-level metadata in many experimental datasets further limits the development of animal-aware validation procedures, which are required to assure biological independence. As a result, most current computational investigations tend to concentrate on binary categorisation (Eghbali et al., 2025; Kumar et al., 2025; Liu et al., 2018; Zou et al., 2021), rather than temporal progression from local histological patches. However, the ability to discern early, intermediate, and late stages directly from high-resolution microscopy images would serve as an advantageous instrument for experimental neuroscience and could increase the reproducibility of tau-related assessments.

Machine learning techniques, particularly CNNs, are ideal for this purpose because they can capture multi-scale spatial representations, subtle texture patterns, and morphological signals across structures such as neuronal somata, neuropil, and cortical layers. Transfer learning architectures, such as InceptionV3 and DenseNet, have shown promising results in small-sample histopathology by using feature representations learnt from large-scale natural image datasets (Hussain et al., 2019; Raghu et al., 2019; Rasa et al., 2024; Shin et al., 2016). When paired with appropriate regularisation and training procedures, these models can be applied to AT8-stained cortical tissue, revealing stage-specific patterns of TBI-induced tauopathy. These characteristics make transfer learning a suitable choice for modelling the temporal development of AT8-positive pathology.

Despite this potential, to our knowledge, no prior study has systematically evaluated whether CNN-based systems can detect the temporal trajectory of tau phosphorylation from 1 day to 3 months post-injury using high-resolution histological patches, representing a major gap in the development of automated stage-resolved tau pathology assessment. Existing methods primarily use binary discrimination, assess total tau burden without progression staging, and analyse whole-slide images rather than field of local views (Signaevsky et al., 2019; Tang et al., 2019; Zou et al., 2021). Automatic staging could speed up experimental workflows, reduce inter-observer variability, improve preclinical intervention screening, and support quantitative digital neuropathology pipelines. These challenges highlight the need for computational approaches capable of distinguishing tauopathy stages directly from high-resolution histological fields.

In this work, we build directly on this gap by evaluating whether deep learning models can perform multi-stage temporal classification of TBI-induced tauopathy using AT8-stained cortical images. Rather than focusing on binary detection or whole-slide quantification, we investigate whether CNN-based architectures can distinguish subtle early, intermediate, and late post-injury stages directly from high-magnification field-of-view patches and evaluate whether the extracted morphological patterns align with known biological trajectories of tau aggregation. This study provides a systematic proof-of-concept for automated temporal staging in experimental neuropathology and lays the foundation for scalable, reproducible deep-learning approaches to characterising tauopathy progression. To our knowledge, this represents the first systematic attempt to determine whether deep learning can reconstruct the temporal progression of TBI-induced tauopathy from small, local histological fields.

## Materials and Methods

### Dataset Description and Image Acquisition

This study’s dataset consists of microscopic images (JPEG or PNG format) of the cerebral cortex of rodents with experimentally induced tauopathy. The images were originally collected at the University of Málaga and were previously analysed in a published study (Edwards et al., 2020). Brain tissue was obtained from P301S tau transgenic mice that had undergone controlled cortical impaction, and coronal sections were immunostained with the AT8 antibody, which detects hyperphosphorylated tau and is a well-established model for studying TBI-induced tauopathy (Braak & Braak, 1995; Edwards et al., 2020; Tran et al., 2011).

All images were captured with a bright-field microscope at high power magnification, which provided enough resolution to see neuronal soma, neuropil, and AT8-positive tau deposits. The actual acquisition metadata (such as objective magnification, pixel size, camera model, or field-of-view dimensions) were not included in the dataset we received and hence cannot be listed here.

The dataset is organised into four temporal classes based on the time elapsed since injury:

- **Class 0:** 1 day post-induction (521 images);
- **Class 1:** 1 week post-induction (122 images);
- **Class 2:** 1 month post-induction (816 images);
- **Class 3:** 3 months post-induction (119 images).

This distribution has a significant class imbalance, with Class 2 over-represented and Classes 1 and 3 under-represented, which encourages the implementation of the data balancing procedures outlined in Sect. 2.2.

The dataset was previously organized by post-injury time point and did not include any animal-level identification, slide IDs, or field coordinates. As a result, the images were treated as independent fields, and per-animal image counts were not possible to determine. All provided fields were incorporated without any further manual filtering or selection.

### Preprocessing and Data Balancing

Each image was pre-processed prior to model training to ensure input consistency across architectures and to enhance model generalisation. All images were resized to 128 × 128 pixels and converted to RGB arrays. Pixel intensities were normalised to the [0,1] range by dividing by 255.

As mentioned earlier, the dataset exhibited a substantial class imbalance (Sect. 2.1.). To mitigate this, minority or underrepresented classes were oversampled using bootstrap resampling, resulting in a balanced training set that did not include synthetic content (He & Garcia, 2009).

To further improve robustness and avoid overfitting, a data augmentation pipeline was used during training. The modifications comprised random rotations (± 20º), horizontal and vertical flips, width and height shifts (up to 20%), zoom perturbations (up to 20%), and minor brightness changes (± 10%). These augmentation strategies were chosen heuristically to imitate true biological heterogeneity in tissue orientation, intensifying staining and sectioning artifacts while preserving the underlying histopathological morphology (Shorten & Khoshgoftaar, 2019; Tellez et al., 2019).

All pre-processing and augmentation procedures were carried out using standard Python libraries for image augmentation and deep learning, ensuring reproducibility and compatibility across all trained architectures. Augmentation was solely applied during training, never on validation images.

### Model Architectures

The present research developed and evaluated three distinct convolutional neural network (CNN) architectures with the goal of classifying microscopic images of cortical tissue depending on the period since tauopathy induction. The approaches are as follows: (1) a custom CNN developed from the ground up, (2) a pre-trained InceptionV3 network adapted by transfer learning, and (3) a pre-trained DenseNet architecture refined using transfer learning. All models were built with the TensorFlow framework (Abadi et al., 2015).

#### Custom CNN (Model 1)

**Fig. 1:**
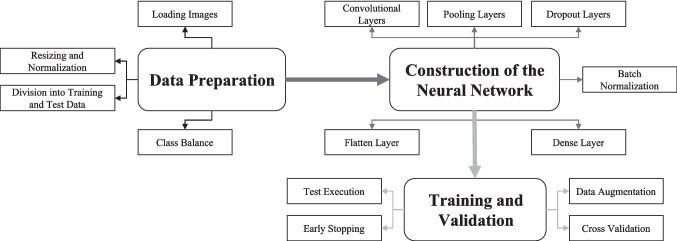
Schematic representation of the custom convolutional neural network (CNN) architecture developed from scratch (Model 1)

The baseline model (Fig. 1) was structured into three convolutional blocks with increasing filter sizes of 32, 64, and 128, each having a 3 × 3 convolution, batch normalization, ReLU (Rectified Linear Unit) activation, and 2 × 2 max pooling (Chan et al., 2020). To reduce overfitting due to the small dataset, a relatively strong dropout rate of 0.5 was used after each block. The resulting feature maps were flattened and processed through a dense layer of 256 ReLU units, followed by a softmax layer that produced four class probabilities. Convolutional and dense layers included L2 regularisation to reduce overfitting (Srivastava et al., 2014). The model was trained from the ground up using the Adam optimizer and sparse categorical cross-entropy loss. The hyperparameters were determined a priori based on preliminary pilot experiments and remained constant during cross-validation. This architecture served as a baseline for determining the dataset's inherent separability prior to implementing transfer learning.

#### InceptionV3 with Transfer Learning (Model 2)

The second model employed the InceptionV3 convolutional architecture (Szegedy et al., 2016), which was pre-trained on ImageNet. InceptionV3's multi-scale feature extraction functionality has shown effective in a variety of medical imaging tasks, including histopathology and radiography (Janowczyk & Madabhushi, 2016). The pretrained base was originally frozen to retain its multi-scale feature extractors, then a custom classification head consisting of global average pooling, a 0.5 dropout rate, and a softmax layer was introduced. Following initial training, the deeper layers were unfrozen for fine-tuning in order to react to tau-stained histological morphology (Tajbakhsh et al., 2016). Batch normalisation layers remained frozen to maintain stable feature statistics during fine-tuning. This strategy leverages the Inception modules' excellent representational capabilities while limiting overfitting in a small dataset.

#### DenseNet with Transfer Learning (Model 3)

**Fig. 2:**
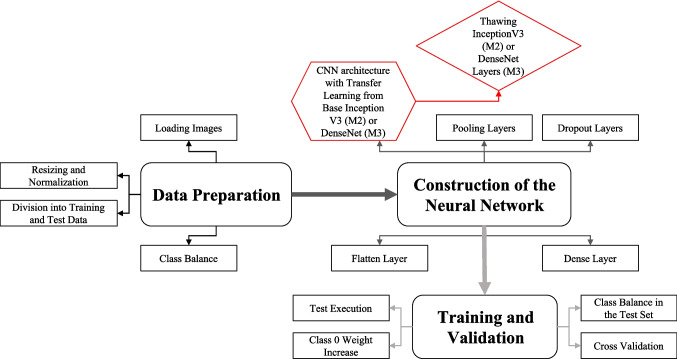
Schematic representation of the InceptionV3 with Transfer Learning (Model 2 – M2) and the DenseNet with Transfer Learning (Model 3 – M3)

The third model relied on the DenseNet architecture (Huang et al., 2017), which was similarly pre-trained with ImageNet. DenseNet's dense connectivity structure encourages feature reuse (Tajbakhsh et al., 2016) and efficient gradient flow, which is useful for biomedical image analysis. Previous histopathology and tissue categorization studies have shown that the design performs exceptionally well (Zhong et al., 2020). As with the previous model, the pretrained model was initially frozen before introducing a custom classification head that included global average pooling, a 0.5 dropout rate, and a softmax layer. The upper layers were then fine-tuned to specialize this model for the target domain while retaining the stability of the pretrained feature extractor. Figure 2 depicts the Model 2 and Model 3 schemes, as well as their parallels with Model 1.

### Training and Validation Strategy

All models were developed in TensorFlow/Keras (Chollet, et al., 2015) and trained using the Adam optimiser with sparse categorical cross-entropy (Kingma & Adam, 2017), which is suitable for multiclass classification with integer labels. Training was carried out using balanced datasets created via bootstrap oversampling within each training fold (Sect. 2.2), which were supplemented with on-the-fly data augmentation to reduce overfitting and increase generalisation.

Hyperparameters such as the learning rate, batch size, dropout rates, and number of epochs were fixed a priori based on preliminary pilot experiments. There was no hyperparameter tuning conducted within the validation folds, preventing information leakage and ensuring that the outcomes reported reflect true internal validation rather than hyperparameter optimisation (Varoquaux 2018).

A k-fold cross-validation scheme was used to estimate the generalisation performance (Kohavi, 1995). The custom CNN (Model 1) was evaluated using a tenfold CV, while InceptionV3 and DenseNet (Models 2 and 3) used a fivefold CV, as initial tests indicated that using higher k-fold values was not beneficial in transfer-learning settings. All splits were stratified to preserve the class distribution in each fold.

For each fold, the models were trained on k-1 subsets and evaluated on the remaining ones. To avoid overfitting, early stopping was implemented based on validation loss (patience = 5–10 epochs depending on the model), and the best-performing model per fold was preserved for metric computation (Prechelt, 1998).

Each model's performance was measured using accuracy, macro F1 score, per-class F1 scores, and one-vs-rest AUC (Saito & Rehmsmeier, 2015). To present statistically grounded estimates, mean values are reported with a 95% confidence interval across folds, rather than single-point estimates. Confusion matrices and ROC curves were also used to investigate class-specific behaviour.

Given the absence of animal-level identifiers in the dataset (Sect. 2.1), the images were treated as independent fields (Roberts et al., 2021), and the evaluation was limited to internal validation. As a result, the metrics presented in this article should not be interpreted as external test performance or animal-level generalisation.

### Explainability Strategy

Since the dataset lacked whole-slide images or spatial metadata, gradient-based interpretability techniques like Grad-CAM could not be used directly (Selvaraju et al., 2020). Instead, explainability was approached conceptually by examining class-specific performance patterns and correlating them to neurobiological characteristics of tauopathy following traumatic brain injury (Tran et al., 2011). The biological interpretation of these patterns is detailed in Sect. 3.

### Pipeline Extensibility

Although this study focused exclusively on fixed-size microscopic fields, the proposed workflow is entirely compatible with more advanced architectures employed in digital histopathology, including patch-based CNNs (Coudray et al., 2018) (e.g., ResNet, EfficientNet), multi-scale models (Rijthoven et al., 2021; Schmitz et al., 2021) (e.g., HookNet, two-stream CNNs) and attention-based encoders (Dosovitskiy et al., 2010) (e.g., Vision Transformers).

First, the present preprocessing and feature extraction method can be modified to include patch-based analysis, which allows for slide-level inference by aggregating predictions from multiple regions (Campanella et al., 2019). Second, the design is inherently extensible to multi-scale models that include both fine-grained cellular features and broader tissue-level context.

Furthermore, the models proposed in this paper are conceptually compatible with Multiple Instance Learning (MIL), a framework commonly employed in whole-slide image classification that does not require instance-level labels (Ilse et al., 2018).

These extensions would make future incorporation into whole-slide imaging (WSI) workflows easier, improve scalability, and allow for deployment on digital neuropathology systems. Although not implement in the present study, pipeline compatibility with these methods encourages future growth beyond field-level prediction.

## Results and Discussion

### Overview and Model Performance

A comparative examination of the three architectures demonstrated a consistent performance hierarchy, with DenseNet outperforming InceptionV3 and Custom CNN. Table 1 summarises the global metrics acquired from cross-validation, including accuracy, macro-F1, macro-AUC, and their respective 95% confidence intervals.

**Table 1:** Summary of overall model performance (mean across folds ± 95% CI)

Model	Accuracy (%)	95% CI	Macro-F1	95% CI	Macro-AUC	95% CI
CNN	65.6	64.5–66.6	0.62	0.60–0.64	0.71	0.69–0.72
InceptionV3	67.7	66.6–69.0	0.65	0.63–0.67	0.74	0.72–0.76
DenseNet	70.9	69.8–71.9	0.68	0.66–0.70	0.77	0.75–0.79

DenseNet outperformed InceptionV3 and the custom CNN on all macro-level metrics. In terms of accuracy, the 95% confidence interval does not overlap with the other models, showing a considerable statistical gain. The confidence intervals for macro-F1 and macro-AUC partialy overlap with InceptionV3, however DenseNet still outperforms InceptionV3 on all metrics. The custom CNN produced a moderate result but maintained stable performance, indicating the dataset's inherent complexity when trained from scratch. InceptionV3 provided an intermediate outcome, owing to its multi-scale feature extraction.

Overall, these results show that transfer learning significantly improves tauopathy progression classification, especially when adopting architectures with strong feature reuse, such as DenseNet.

### Per-Class Performance

The F1 scores by class (Table 2) show a significant variation in classification difficulty between the four stages of tauopathy progression. All three structures follow a generally consistent pattern, with the 1-week class (Class 1) having the highest discriminability and the 1-day class (Class 0) the lowest.

**Table 2:** Mean per-class F1-scores for the three models

Class	CNN	InceptionV3	DenseNet
0	63%	51%	26%
1	69%	92%	95%
2	55%	54%	63%
3	81%	74%	71%

#### Early Stage (Class 0–1 day)

Class 0 performance was poor across all architectures (63% for CNN, 51% for InceptionV3, and 26% for DenseNet). This may be rationalised by the low AT8-positive signal observed 24 h after injury, when phosphorylated tau is sparse and morphological signs are faint (Edwards et al., 2020; Hoshino et al., 1998). The limited separability of Class 0 is biologically plausible, as early post-injury tissue closely resembles baseline neuropil, as reported in early post-injury histology in P301S/TBI models (Yoshiyama et al., 2007).

#### Acute Stage (Class 1–1 week)

Class 1 was the most accurately identified class, with F1 scores of 95% (DenseNet), 92% (InceptionV3), and 69% (CNN). This finding is biologically supported: one week after injury, the cortex shows substantial AT8-positive perisomatic aggregates and numerous neuropil threads, resulting in a robust and spatially coherent histopathological signature (Edwards et al., 2020; Yoshiyama et al., 2007). Consistent with this, several TBI models show a significant rise in tau abnormalities throughout this interval (Tran et al., 2011). As a result, DenseNet obtained a very good separability for this class.

#### Intermediate Stage (Class 2–1 month)

Class 2 performed averagely (55% for CNN, 54% for InceptionV3, and 63% for DenseNet). Misclassifications were most common with Class 3, which is biologically compatible with the gradual, rather than sudden, increase of tau pathology one to three months after injury (Clavaguera et al., 2009; Edwards et al., 2020; Yoshiyama et al., 2007). AT8 positive tau is certainly present in this intermediate stage, but its distribution is heterogeneous and spatially variable, resulting in less stable morphological cues and contributing to inconsistent predictions (Arendt et al., 2016; Edwards et al., 2020; Tran et al., 2011).

#### Late Stage (Class 3–3 months)

F1-scores for Class 3 varied from 81% (CNN) to 74% (InceptionV3) and 71% (DenseNet), indicating moderate discriminability. This behaviour reflects the underlying biology: by three months, tauopathy is widespread but still morphologically overlapping with the disease reported at one month, indicating a mostly quantitative rather than qualitative development (Clavaguera et al., 2009; Edwards et al., 2020). Unlike the extremely identifiable AT8-positive patterns found after one-week, later stages lack unambiguous class-specific structural motifs, and their heterogeneous distribution lowers the confidence of CNN-based predictions (Arendt et al., 2016; Yoshiyama et al., 2007).

When examined together, the per-class performance reveals a consistent biological trend. The highest separability was seen at one week, which corresponds to the time when AT8-positive tau pathology becomes most prominent and morphologically distinct (Edwards et al., 2020; Tran et al., 2011; Yoshiyama et al., 2007). In contrast, the lowest separability was seen after one day, which is consistent with the near absence of phosphorylated tau following injury (Edwards et al., 2020; Hoshino et al., 1998; Tran et al., 2011). The intermediate difficulty in separating 1-month and 3-month samples reflects the gradual and mainly quantitative evolution of tauopathy in later stages, when At8-positive inclusions increase in burden while sharing structural features (Arendt et al., 2016; Clavaguera et al., 2009; Edwards et al., 2020). These findings suggest that the models accurately captured important features of the temporal dynamics of tau accumulation over post-injury intervals.

### Confusion Matrixes and Class Separability

**Fig. 3:**
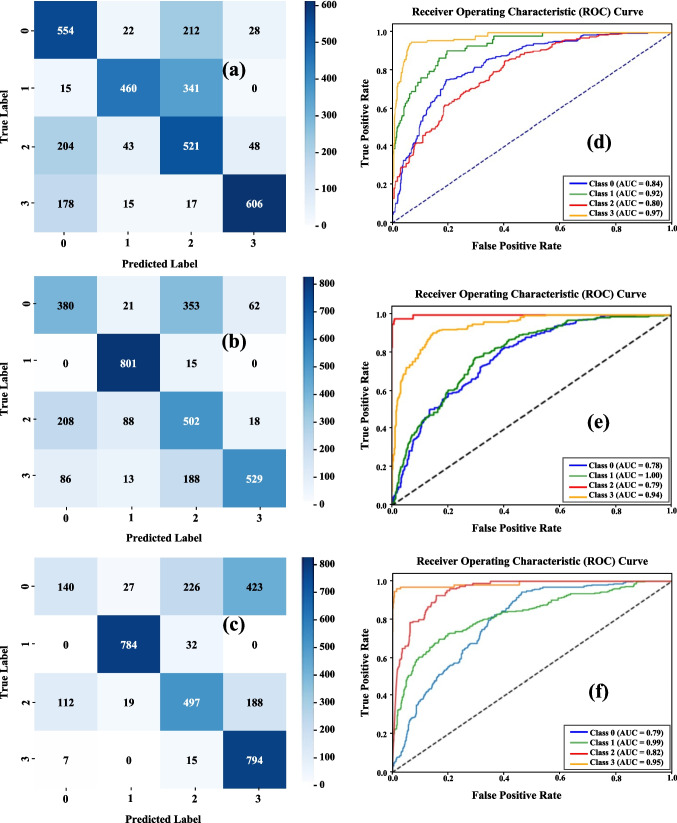
Comparative performance of: (a–c) Confusion matrices for the CNN, InceptionV3, and DenseNet models, respectively; and (d–f) Corresponding class-wise ROC curves (with AUC values) for the CNN, InceptionV3, and DenseNet models

The confusion matrices (Fig. 3 (a), (b) and (c)) for the three architectures (CNN, InceptionV3, and DenseNet) show consistent misclassification patterns which follow the biological characteristics of tau pathology across time, particularly for transfer-learning models. Although the total number of correct predictions varies amongst models, the underlying error pattern is similar, with CNN exhibiting more architecture-driven confusion patterns, likely due to its limited representational capacity.

In both transfer models (InceptionV3 and DenseNet), Class 1 (1 week) demonstrated the highest separability, with strongly concentrated diagonal entries and minimal cross-class confusion. This aligns with the marked increase in AT8-positive perisomatic aggregates and neuropil threads typically observed at this temporal stage (Edwards et al., 2020). In contrast, the baseline CNN did not capture these discriminative features as efficiently, demonstrating higher confusion among all classes, including 1 week.

In contrast, Class 0 (1 day) is inconsistently recognised. The CNN model has the highest true-positive count (554), whereas InceptionV3 and DenseNet have a higher distribution of Class 0 data into Classes 2 and 3. This reflects the naturally weak and diffuse AT8 signal at 24 h, which lacks the structured morphology that deeper models expect. As a result, the DenseNet and InceptionV3 models, which depend more heavily on prominent and multi-scale patterns, frequently misread weak early stage staining as more advanced stages (most often Class 2).

Confusion between Classes 2 (1 month) and 3 (3 months) exists in both transfer-learning models, although in an asymmetrical manner: DenseNet misclassifies some 1-month images as 3-month images, whereas InceptionV3 exhibits the opposite trend. In the baseline CNN model, the confusion is minimal. This behaviour is consistent with the biological similarity between the two stages, since both show extensive AT8-positive neuritic degeneration, with the differences being mostly quantitative rather than qualitative (Edwards et al., 2020; Yoshiyama et al., 2007).

DenseNet displayed strong diagonal performance, especially in Class 3 (3 months), showing strong separability for late-stage pathology. InceptionV3 performs comparably, with the maximum correct classification count for Class 1 (1 week), but with slightly higher dispersion in Classes 2 and 3. In contrast, the custom CNN shows the most diverse confusion patterns, particularly between the early (0–1) and advanced (2–3) stages.

Overall, the confusion matrices show that much of the difficulty in classification is due to the underlying biological progression of tauopathy: a weak or non-existent AT8 signal after 1 day (Edwards et al., 2020; Hoshino et al., 1998; Tran et al., 2011), a clear morphological peak after 1 week driven by dense perisomatic and neuropil AT8-positive pathology (Edwards et al., 2020; Yoshiyama et al., 2007), and a more gradual quantitative difference between 1 and 3 months (Clavaguera et al., 2009; Edwards et al., 2020). This trend is more visible in transfer learning models (InceptionV3 and DenseNet), where misclassifications correlate with the predicted temporal structure of tau aggregation. However, the basic CNN model's insufficient ability to extract subtle mid- to late-stage features cause certain non-biologically driven errors.

### ROC Curve Analysis

The ROC curves (Fig. 3 (d), (e) and (f)) provide additional insight into class separability across the three architectures. Consistent with previous analyses, all models show that Class 1 (1 week) and Class 3 (3 months) have higher AUC values due to their prominent AT8-positive morphological fingerprints (Edwards et al., 2020; Tran et al., 2011; Yoshiyama et al., 2007). DenseNet and InceptionV3 have comparable ROC curve profiles, with DenseNet achieving somewhat better AUCs at the later stages and InceptionV3 at the most pronounced stage (1 week).

Class 0 (1 day) has the lowest AUC value among transfer learning models (InceptionV3: 0.78, DenseNet: 0.79), whereas the CNN model has the lowest value for Class 2 (0.80 vs 0.84 for Class 0). Nonetheless, this is consistent with the low AT8 immunoreactivity observed after 24 h of injury, where tau phosphorylation is weak or absent (Edwards et al., 2020; Hoshino et al., 1998; Tran et al., 2011).

In intermediate-stage tauopathy (Class 2—1 month), all architectures show moderate AUC values (0.79–0.82), indicating progressive but partially overlapping tau deposition (Clavaguera et al., 2009; Edwards et al., 2020).

Overall, ROC patterns show that separability increases when tau pathology is either strongly expressed (1 week, 3 months) or has a distinct structural organisation. These patterns are qualitatively consistent with the reported temporal dynamics of AT8-positive tau accumulation following TBI and in P301S tauopathy models.

### Comparative Statistical Analysis

To compare the three architectures, accuracy and macro-F1 values were analysed across the cross-validation folds for each model. DenseNet consistently achieved higher fold-level values compared to InceptionV3 and custom CNN, which is consistent with the global metrics reported in Sects. 3.1 and 3.2. The 95% confidence intervals calculated from the fold distributions indicate minimal overlap between DenseNet and the other models, suggesting a meaningful performance advantage with the internal validation setting.

Although no formal statistical test was conducted, the fold-wise distributions show a clear separation between DenseNet and the other architectures, whereas CNN and InceptionV3 have more similar variability. These descriptive trends corroborate DenseNet's superior stability and discriminative capabilities.

Importantly, these comparisons represent relative internal performance rather than conclusive evidence or overall superiority. Since neither animal-level IDs or external validation were provided, fold-level estimates could capture biological or acquisition-related variability. Extensive external testing will be required to establish whether the reported performance hierarchy holds true across diverse staining intensity, imaging settings, and biological samples.

### Neurobiological Contextualization of Model Behaviour

#### Interpretation of Classification Behaviour Across Temporal Stages

The per-class performance patterns reported across the three architectures substantially are consistent with documented biological aspects of tauopathy development after traumatic brain injury. The 1-week class (Class 1) has high discriminability, which corresponds to the typical increase of AT8-positive immunoreactivity seen at this stage. Around one week after injury, hyperphosphorylated tau accumulates in perisomatic aggregates, neuropil threads, and discrete laminar patterns, particularly in cortical layers II/III (Arendt et al., 2016; Edwards et al., 2020; Yoshiyama et al., 2007). These well-defined and spatially consistent morphological signals provide a strong visual signature, allowing deep learning models, particularly DenseNet and InceptionV3, to attain F1 scores of around or above 0.90 in this class.

In contrast, Class 0 (1 day) was consistently challenging across models. At 24 h post-injury, AT8-positive tau is usually sparse or absent, with only subtle background staining and little to no organised deposits or neuritic pathology (Edwards et al., 2020; Hoshino et al., 1998; Tran et al., 2011). As a result, numerous images resemble non-pathological cortex. This weak signal explains why all architectures struggle to differentiate this class, and why deeper, more regularised DenseNet has particularly low sensitivity: deeper architectures prioritise more prominent and higher-order patterns, potentially underfitting extremely small morphological cues.

The intermediate performance of Class 2 (1 month) and the confusion shared with Class 3 (3 months) demonstrate the gradual and continuous nature of tau accumulation in later stages. Between one and three months after injury, AT8-positive pathology becomes more pervasive, although the changes are primarily quantitative rather than qualitative: both time periods show severe neuritic degeneration and diffuse cortical tau deposition (Clavaguera et al., 2009; Edwards et al., 2020; Jucker & Walker, 2011). This results in overlapping morphological profiles, and all models achieve mid-range F1 scores for these stages, with frequent misclassifications between them, especially in transfer-learning models.

When combined, the categorisation behaviour of the three models resembles important neuropathological trajectories of tauopathy rather than arbitrary error patterns. Rather than spurious artefacts, the models appear to rely on meaningful biological features, such as strong, focal AT8 immunoreactivity at one week, weak or absent early signal at one day, and progressive yet overlapping pathology at later stages. This adds to the biological plausibility of the learnt representations and shows that many model errors occur at periods where the underlying pathology is itself difficult to distinguish, even for experienced observers.

#### Mechanistic Links between Traumatic Brain Injury, Microbleeds, Iron Toxicity and Tau Aggregation

Traumatic brain injury initiates a series of secondary pathological processes that extend well beyond the original trauma mechanism. Among the most significant are microvascular disruption, microbleeds, and iron deposition, which create a biochemical environment that promotes tau phosphorylation, aggregation, and neurodegeneration over time. Blood–brain barrier disruption and microhaemorrhages release haemoglobin, heme, and iron into the parenchyma, causing oxidative stress, mitochondrial dysfunction, and chronic microglial activation, according to experimental and clinical research (Christodulou et al., 2025a). Excess iron catalyses free radical production and increases neuronal vulnerability, which exacerbates the neurotoxic effects of phosphorylated tau, accelerating synaptic dysfunction and cortical atrophy.

These haemorrhage-associated processes develop slowly, creating a progressive inflammatory and iron-rich environment that lasts for weeks to months after TBI. Chronic iron-driven inflammation is becoming recognised as the mechanistic link between TBI and long-term tauopathy, with microbleeds and hemosiderin deposits acting as focal initiators of persistent neurogenerative signalling (Christodulou et al., 2025b). This framework is consistent with observations in repetitive or blast related TBI, in which iron buildup and microvascular pathology worsen tau seeding, aggregation, and cortical degeneration (Christodulou et al., 2025c).

Within this mechanistic context, our models' classification behaviour can be biologically interpretable. The low separability at 1 day is most likely due to the fact that microbleed-related iron toxicity and inflammatory cascades have only recently begun, with no detectable AT8-positive aggregates. The strong discriminability at 1 week corresponds to the onset of significant tau phosphorylation, which is consistent with the early effects of iron-mediated oxidative stress. Finally, the partial overlap between the 1- and 3-month stages shows the chronic, gradual nature of iron-associated neurodegeneration, in which tau accumulation increases but remains morphologically continuous rather than sharply distinguished.

When considered together, microbleeds, iron deposition, and inflammation could provide a mechanistic explanation for both the biological trajectory of tauopathy after TBI and the class-specific patterns observed in our deep learning models.

### Error Analysis

A comparison of misclassification patterns across all models reveals that the errors were not random, but rather followed biologically relevant tendencies. The most common source of errors was confusion between one month (Class 2) and three months (Class 3), which is consistent with the progressive and continuous nature of tau aggregation in P301S tauopathy models. Both stages show widespread AT8-positive neuritic degeneration, with more quantitative than qualitative differences (Clavaguera et al., 2009; Edwards et al., 2020; Jucker & Walker, 2011). As a result, even the most regularised architectures, such as DenseNet, exhibited residual ambiguity between these two classes.

A second recurring pattern was that transfer-learning models, particularly DenseNet, misclassified early-stage images (Class 0—1 day) as later stages, most commonly Class 2 or Class 3 in the transfer-learning models. This behaviour is biologically plausible: 24 h after damage, AT8 immunoreactivity is weak or non-existent, resulting in images that closely resemble the baseline cortex (Edwards et al., 2020; Hoshino et al., 1998; Tran et al., 2011). Deep architectures that prioritise strong and coherent morphological characteristics may thus underfit the highly delicate cues available at this time period, resulting in a systematic overestimation of disease progression.

In contrast, errors involving Class 1 (1 week) were uncommon, especially in transfer learning models. This class has both strong F1-scores (except for the CNN model) and steep ROC curves, with misclassification occurring primarily in borderline cases with unusually low AT8 signal. This finding confirms that model performance is directly related to the intrinsic morphological expressiveness of each pathological stage.

Overall, the observed error patterns suggest that model performance is influenced more by the biological structure of the dataset than by architectural differences. Misclassifications occurred most frequently when the underlying pathology was either (i) too subtle to reliably distinguish from baseline tissue (Class 0) or (ii) too continuous to be accurately separated (Classes 2 and 3). These findings indicate that, rather than purely architectural improvements, additional contextual information, such as patch aggregation, slide-level metadata, or multimodal inputs, will be required to achieve further performance gains.

### Comparison with Related Deep Learning Approaches in Tau Pathology

Although no previous research has focused on multi-stage temporal classification of TBI-induced tauopathy using AT8-stained histology, our findings can be contextualised within relevant deep-learning approaches in tau pathology.

Signaevsky et al. (Signaevsky et al., 2019) and Tang et al. (Tang et al., 2019) demonstrated that CNNs may quantify tau burden and accurately distinguish Alzheimer's disease pathological subtypes, however these models relied on static snapshots rather than temporally structured progression. Similarly, Wang et al. (Wang et al., 2024) demonstrated that CNNs are effective at capturing fine cortical microarchitecture, while Zou et al. (Zou et al., 2021) found that tau-PET diagnostic value increased with deep learning.

Compared to existing approaches, our framework addresses more subtle morphological differences and a more difficult four-stage classification problem. The present study's performance hierarchy (DenseNet > InceptionV3 > Custom CNN) is consistent with previous findings that connected or multiscale networks outperform in small-sample histopathology tasks (Hussain et al., 2019; Raghu et al., 2019; Shin et al., 2016). Thus, while conceptually similar to existing tau-focused models, our study goes beyond them by demonstrating that deep learning can track the temporal trajectory of tau accumulation after TBI, a task not addressed in earlier computational pathology research.

### Practical Implications for Digital Neuropathology

The study's findings emphasise the potential of deep learning models to assist in the automated staging of tauopathy progression in AT8-stained histological sections following traumatic brain injury. Although the current dataset consists of small, field-of-view patches rather than whole-slide images, the consistent performance hierarchy observed across architectures suggests that such models could form the basis for more comprehensive digital neuropathology pipelines.

In practice, a system capable of reliably distinguishing early, acute, and late stages of tauopathy may assist neuropathologists by providing rapid pre-screening of large volumes of tissue, prioritising regions with high pathological burden, and identifying subtle early-stage abnormalities that would normally necessitate extensive manual examination. This is especially important in experimental studies of TBI, where lesion distribution varies and slide counts are frequently high (Edwards et al., 2020).

Automated stage prediction can further enhance quantitative analysis by allowing for objective comparisons across animals, time points, and experimental conditions. Consistent predictions across folds and architectures lay the groundwork for downstream applications such as lesion mapping, temporal progression modelling, and the correlation of histopathological severity with behavioural, molecular, or imaging readouts, which is common in preclinical tauopathy research (Clavaguera et al., 2009; Yoshiyama et al., 2007).

Although further improvement is necessary for deployment, such as whole-slide processing, patch aggregation strategy integration, explainability maps, and external validation, the results show that deep learning can extract important biological patterns from AT8-stained cortex. With further refinement, such systems could potentially offer semi-automated workflows for tauopathy assessment, increasing consistency, lowering manual workload, and accelerating analysis in both experimental research and translational neuropathology.

### Limitations

Several limitations of the current investigation should be addressed. First, the dataset is made up of small, high-magnification fields of view with no slide or animal IDs, making it impossible to enforce animal-level separation between folds. As a result, it is impossible to assure that images of the same animal were not spread throughout multiple splits, limiting the ability to examine inter-animal variability. Although cross-validation was rigorously stratified and hyperparameters were fixed a priori to decrease the chance of leakage, the lack of entirely independent test subjects means that the outcomes reported should be considered as internal validation rather than external generalisation.

Second, every image originates from a single staining protocol (AT8) and a single experimental centre. As a result, the models were not affected by variations in staining intensity, tissue processing, scanner features, or laboratory-specific artefacts, all of which have been shown to have a significant impact on deep learning performance in histopathology. Before any translational deployment can take place, more extensive multi-centre validation is required.

Third, current models only function at the patch/FOV level, with no access to multiscale spatial context or slide-level aggregation. This limits the ability to track long-term laminar organisation, regional gradients, or tau deposition continuity, all of which are important features for intermediate or late stages with heterogeneous pathologies. This constraint could be addressed by approaches such as multiple-instance learning or hierarchical attention, which integrate fine- and coarse-scale information.

Finally, the study did not use explainability techniques like Grad-Cam or saliency mapping. The dataset's small field-of-view images limit the use of such approaches, but explicit visual explanations are still required for interpretability, transparency, and user confidence. Future work that incorporates whole-slide images, spatial metadata, and model-agnostic interpretability tools will be required to improve the system's clinical and experimental interpretability.

### Future Work

Future work will focus on expanding the current framework to include slide- and animal-level modelling, addressing the absence of global contextual information inherent in patch-based analysis. Multiple-instance learning (MIL), hierarchical attention networks, and transformer-based architectures could integrate both local and global morphological signals, improving discrimination between intermediate and late stages of tauopathy. Patch-aggregation techniques and multi-scale encoders may also aid in capturing laminar organisation and long-range spatial continuity of pathology.

Another objective is the implementation of model interpretability tools. When whole-slide images or high-resolution spatial metadata are available, approaches like Grad-Cam, integrated gradients, or activation maximisation can be used to uncover the morphological substrates that drive the model's predictions and facilitate expert neuropathological validation.

External validation is a necessary next step. To determine generalisability and robustness, the models will need to be tested on multi-centre datasets with varying staining intensity, tissue preparation, scanner hardware, and animal cohorts. Harmonization procedures such as stain normalization, domain adaptation, or self-supervised pre-training on large unlabelled histological corpora can further improve cross-centre stability.

Finally, integrating the current framework with quantitative readouts, such as automated tau-load estimation or spatial mapping of degeneration, may increase its practical relevance. These advancements would together enhance the interpretability, robustness, and translational potential of the technique, opening the door for semi-automated tools to support digital neuropathology in experimental and preclinical research.

## Conclusions

Deep learning models can reliably characterise the temporal progression of tauopathy in AT8-stained cortical tissue following traumatic brain injury. Across architectures, the networks captured biologically meaningful morphological differences, with DenseNet providing the most robust and consistent performance. The observed patterns, such as limited separability at 24 h, a clear discriminative peak at one week, and progressive overlap between one and three months, were coherent with established trajectories of hyperphosphorylated tau accumulation.

Beyond stage classification, these findings highlight the potential of deep learning to support digital neuropathology workflows through automated staging, pre-screening and quantitative assessment of tau burden. However, the current results reflect internal validation on patch level data without animal-level identifiers or external cohorts, and should therefore be interpreted as preliminary.

Future research should prioritise slide-level or multi-scale modelling, explainability techniques such as Grad-CAM, and multi-centre datasets for assessing generalisability. Integrating histological predictions with behavioural or molecular biomarkers may further advance translational applications.

Overall, this study provides a solid basis for applying deep learning in the quantitative analysis of TBI-induced tauopathy, and it represents a step towards more objective and scalable neuropathological assessment.

## Data Availability

The histological image dataset used in this study was obtained from previously published experimental work and is not publicly available due to institutional restrictions and the absence of animal-level identifiers. The data may be made available from the corresponding author upon reasonable request and with permission from the original data providers. All code used for model training and analysis may be available upon reasonable request.
